# Cardiovascular Magnetic Resonance Reveals Cardiac Pathophysiology in Autoimmune Rheumatic Diseases

**DOI:** 10.31138/mjr.32.1.15

**Published:** 2021-03-31

**Authors:** George Markousis-Mavrogenis, Petros P. Sfikakis, Loukia Koutsogeorgopoulou, Theodoros Dimitroulas, Gikas Katsifis, Aikaterini Giannakopoulou, Paraskevi Voulgari, Genovefa Kolovou, George D. Kitas, Sophie I. Mavrogeni

**Affiliations:** 1 Onassis Cardiac Surgery Center, Athens, Greece; 2 Joint Rheumatology, Laikon Hospital, Athens, Greece; 3 Department of Pathophysiology, Laikon Hospital, National Kapodistrian University of Athens, Athens, Greece; 4 Department of Internal Medicine, Aristotle University of Thessaloniki, Thessaloniki, Greece; 5 Naval Hospital, Athens, Greece; 6 Agia Sophia Children’s Hospital, Athens, Greece; 7 Rheumatology Department, University of Ioannina, Ioannina, Greece; 8 Arthritis Research UK Epidemiology Unit, Manchester University, Manchester, United Kingdom

**Keywords:** Cardiovascular magnetic resonance, myocardial perfusionfibrosis, coronary artery disease, vasculitis, myocardial inflammation

## Abstract

**Background/Aims:**

The high incidence of cardiovascular disease (CVD) in patients with autoimmune rheumatic diseases (ARDs) is the main driver towards increased mortality in this patient group. Cardiovascular magnetic resonance (CMR) can non-invasively and robustly detect CVD in ARD patients at an early stage of development. The review summarises the diagnostic information provided by CMR in ARD patients.

**Summary:**

CMR uses a strong magnetic field combined with radio-frequency pulses (pulse sequences) to generate images. Firstly, balanced steady-state free precession (bSSFP) can be used for evaluating cardiac anatomy, mass, wall motion, atrial/ventricular function. Secondly, T2-weighted imaging (T2-W) can be used for oedema detection, which appears as a high signal intensity area on STIR (short tau inversion recovery) images. T2 mapping is a newer T2-W technique that can provide more optimal identification of myocardial oedema. Lastly, late gadolinium enhanced (LGE) T1-W images, taken 15 min. after injection of contrast agent, allow the detection of myocardial replacement fibrosis, which appears as a bright area in a background of black myocardium. However, LGE has inherent disadvantages for the assessment of diffuse myocardial fibrosis. Therefore, T1 mapping and extracellular volume fraction (ECV) have been developed to quantify diffuse myocardial fibrosis.

**Results:**

Although multicentre studies are still missing, the CMR parameters have been extensively applied for the identification of oedema/fibrosis and treatment decision making in ARDs.

**Conclusions:**

Tissue characterisation with CMR allows early and robust identification of CVD in ARD patients and contributes to personalized management in the patients.

## INTRODUCTION

Autoimmune rheumatic diseases (ARDs) are a collection of heterogeneous diseases in which tolerance to self-antigens and/or immunoregulatory mechanisms become compromised, thus leading to inappropriate immune reactivity against body tissues. The mainstay of therapy is immunomodulatory treatment as to prevent inappropriate immune activation. Although new targeted treatments currently available for the management of ARDs have resulted in significant reductions of disease-associated mortality, patients with ARDs still have a lower average life expectancy compared with the general population,^1^ mainly due to the increased incidence of cardiovascular disease (CVD).^2–6^

CVD in patients with ARDs can be caused by various pathophysiologic phenomena. These include systemic and/or cardiovascular inflammation, perfusion defects due to micro-/macro-vascular coronary artery disease (CAD), abnormal vasoreactivity, myocardial fibrosis, coagulation abnormalities, pulmonary hypertension due to cardiac/pulmonary involvement, valvular diseases, and effects of immunomodulatory medication.^7,8^ Irrespective of aetiology, CVD in this patient population usually presents asymptomatically or with few subtle symptoms, which are often overlooked or written off as constitutional symptoms. Clinically overt CVD presents late in the course of ARDs and carries a poor prognosis, as it indicates advanced disease progression and/or decompensation.^9^ Therefore, robust and early identification of patients with ARDs that develop CVD, as well as appropriate treatment to prevent disease progression and reduce morbidity and mortality are essential for improving prognosis in this patient group.

Regarding the early identification of patients with ARDs and CVD, many strategies have been investigated including clinical examination, circulating biomarkers, and cardiovascular imaging modalities. Cardiovascular magnetic resonance imaging (CMR) has emerged as the most effective diagnostic modality for this purpose, as it can evaluate cardiac function and characterize myocardial tissues with regard to oedema/fibrosis in the same examination without making use of ionizing radiation.^10,11^ The aim of this review is to present a concise summary of the diagnostic information that can be provided by the complete array of CMR-generated images in patients with ARDs, and to discuss their clinical significance in the context of the early detection of CVD in these patients.

## BASICS OF CARDIOVASCULAR MAGNETIC RESONANCE

The greatest advantage of CMR lies in that it can provide direct information about the status of all cardiac tissues in a non-invasive manner and without employing ionising radiation.^10^ In contrast to echocardiography, image acquisition with CMR is operator-independent and has excellent reproducibility.^10^ This is because images are acquired by using a strong magnetic field and a sequence of radio frequency photon pulses (so-called pulse sequences), which are not limited by parameters such as sufficient acoustic window, as in the case of echocardiography. The basic pulse sequences that are used in the clinical setting include^10^ balanced steady-state free precession (bSSFP), as well as a variety of T1-weighted (T1-W) and T2-weighted (T2-W) sequences. We have previously described the basic physics behind CMR as well as how each pulse sequence functions in detail.^10,11^ These will now be presented with less emphasis on technical details and more focus on their clinical utility, combined with illustrative example images for each one. As a side note, since CMR uses a strong magnetic field to generate images, the strength of said magnetic field is measured in Tesla units (abbreviated as T); currently, most CMR scanners operate using a magnetic field strength of 1.5 or 3 T.

## PULSE SEQUENCES AND THEIR CLINICAL SIGNIFICANCE

### Balanced Steady-state Free Precession (bSSFP)

**Figure 1.**
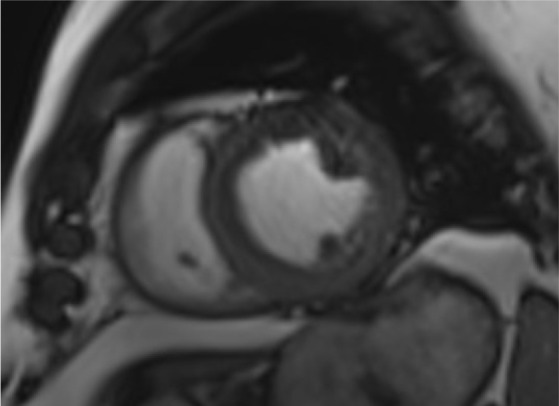
Short axis bSSFP for ventricular function evaluation.

Balanced steady-state free precession at a magnetic field strength of 1.5 Tesla is considered the gold standard for characterising cardiac anatomy, myocardial mass, wall motion, atrial, and ventricular function of both the left and right ventricles (LV/RV) (**Figure 1**).^12^

### T2-Weighted (T2-W) Imaging

**Figure 2.**
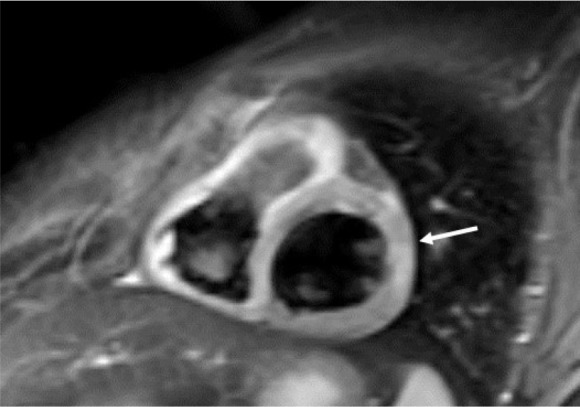
Short axis STIR showing localised, subepicardial oedema, due to autoimmune myocarditis oedema.

Acquisition of these images is based on the prolongation of the transverse relaxation time (T2) caused by water accumulation due to oedema.^13,14^ Oedema represents the acute myocardial reaction to any kind of damage, be that ischemic or inflammatory. Oedema may be localised (**Figure 2**) or diffuse, subendocardial or transmural following the territory of coronary arteries as in CAD, subepicardial as in myocarditis (**Figure 2**) and diffuse subendocardial as in vasculitis.

### Short tau inversion recovery (STIR)

The pulse sequence originally developed for the identification and quantification of myocardial oedema is named short tau inversion recovery (STIR). Oedema appears as a high signal intensity area on images derived using the STIR sequence, where the signal from fat and the blood pool is suppressed to improve the contrast between oedema, normal myocardium, and the LV cavity. However, the utility of STIR images may be limited by poor contrast between healthy and oedematous areas, high dependency on magnetic field homogeneity, loss of signal due to cardiac motion, subendocardial slow flow hyperintensity, susceptibility to motion artifacts, and subjective visual interpretation by different readers.^15,16^

### T2 Mapping

To overcome these limitations of STIR, a new imaging approach called T2 mapping has been developed. T2 mapping is a technique used to construct a map of the myocardium based on the individual T2 value of each voxel. At a magnetic field strength of 1.5 Tesla, the mean and standard deviation of T2 mapping in the myocardium of healthy adults was 52±3ms in a study by Giri et al. in 14 participants, and 55±5ms in a study by Wassmuth et al. in 73 participants. These values are independent of body surface area and/or heart rate and have excellent reproducibility.^17,18^

### T1-Weighted (T1-W) Imaging

The T1 relaxation time is a key parameter of soft tissue contrast in MRI.^19^ Similar to T2-W imaging, acquisition of T1-W images is based on the prolongation of the longitudinal relaxation time (T1). This can be caused by expansion of the extracellular space as occurs in the case of deposition of extracellular matrix as part of myocardial fibrosis, or volume shift from the intravascular to the extravascular compartment due to inflammatory processes, leading to increased vascular permeability.^19^ Different T1-W pulse sequences have different sensitivities to these processes as described below.

### Late gadolinium enhancement (LGE)

**Figure 3.**
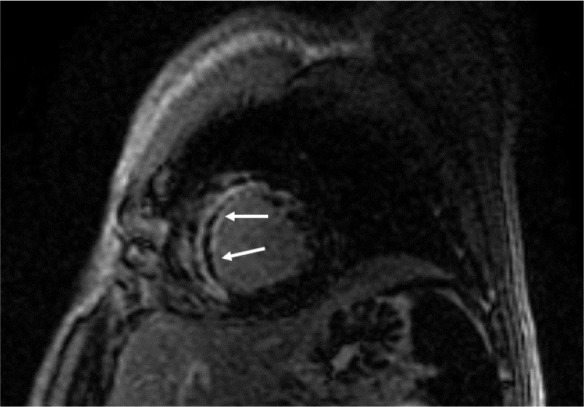
Short axis T1-W image showing transmural LGE in the anteroseptal wall of LV, due to left anterior descending coronary artery obstruction (white area). The black area within the white area represents microvascular obstruction.

**Figure 4.**
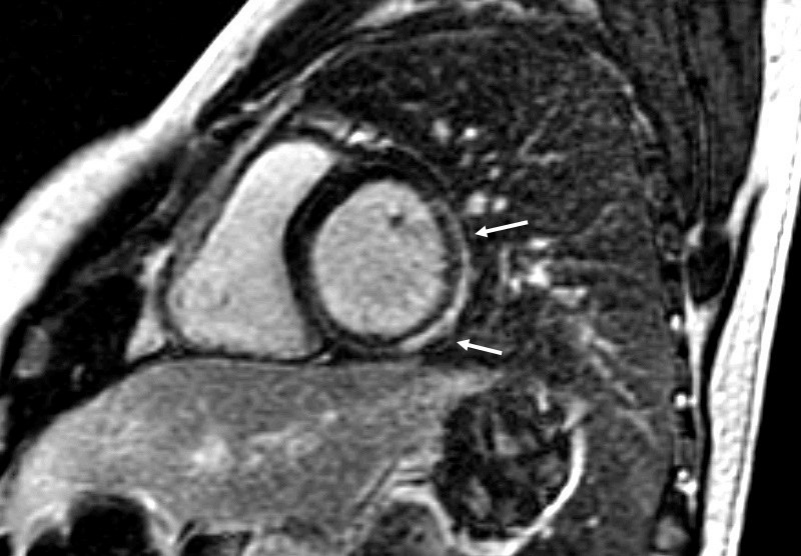
Short axis T1-W image showing subepicardial LGE in the lateral wall of LV, due to autoimmune myocarditis.

**Figure 5.**
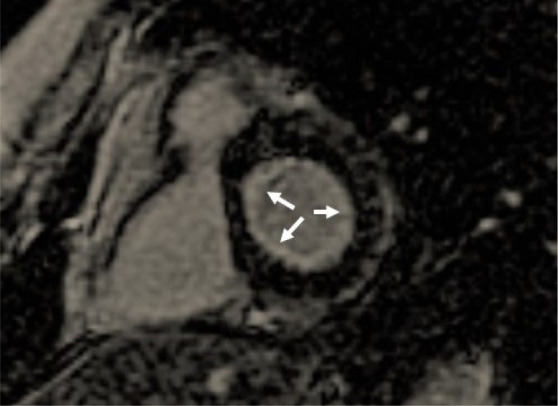
Short axis T1-W image showing diffuse subendocardial LGE indicative of diffuse subendocardial fibrosis, due to small vessel vasculitis.

Late gadolinium enhanced T1-W images (LGE) obtained using standardized pulse sequences 15 min. after the infusion of paramagnetic gadolinium-based contrast agent, allow the detection of myocardial fibrotic tissue (scar).^10^ Standard gadolinium-based contrast agents are distributed throughout the extracellular space and shorten T1 relaxation times of myocardium proportional to the local concentration of gadolinium. Areas of fibrosis and scar will therefore exhibit shorter T1 relaxation times, in particular, after contrast administration. This appears as a bright area in a background of nulled, black myocardium, giving rise to the characteristic pattern of “bright is dead”.^10^ According to the type and location of LGE, the cause of the fibrosis could be attributed to CAD if the lesion is subendocardial, or transmural along the distribution of the coronary arteries (**Figure 3**). In contrast, subepicardial or patchy LGE usually in the inferolateral wall is characteristic of any kind of myocarditis (**Figure 4**). Finally, a diffuse subendocardial pattern of fibrosis is typically seen in small vessel disease, as in the case of systemic sclerosis, antiphospholipid syndrome, rheumatoid arthritis and small vessel vasculitides (**Figure 5**).^10^

### Angiography with T1-W Imaging

T1-W imaging after pharmacologic stress with adenosine and bolus injection paramagnetic contrast agent can provide an accurate and reproducible evaluation of myocardial perfusion during stress.^10^ This approach allowed the early detection of perfusion defects in patients with systemic sclerosis not experiencing any cardiovascular symptoms,^20^ and those with antiphospholipid syndrome.^21^ Lastly, by injecting a bolus of paramagnetic contrast agent, it is possible to perform non-invasive angiography, which can provide important information about great vessel patency and mural inflammation in great vessel vasculitides.^10^

### T1 Mapping

Although LGE is well-validated as the technique of choice for the detection of focal myocardial scars (replacement fibrosis), it has inherent limitations with regard to the assessment of diffuse myocardial fibrosis, as it is based on the signal intensity differences between scarred and normal myocardium to generate image contrast. Since a normal myocardial reference value is required for the LGE images, this approach is unlikely to detect diffuse fibrosis if there is no clear distinction between fibrotic tissue and normal myocardium, as is often the case in patients with ARDs.^22,23^ To overcome this limitation, a CMR imaging technique called T1 mapping has been developed. T1 mapping can be measured without paramagnetic contrast agent (native or pre-contrast T1 mapping) and after administration of paramagnetic contrast agent (post-contrast T1 mapping). Similar to T2 mapping, T1 mapping provides a quantitative assessment of tissue characterization and enables identification of early myocardial fibrosis, which is otherwise undetectable by currently used circulating biomarkers.^24^ The mean and standard deviation of T1 mapping values in healthy volunteers are 995.8±30.9ms and 1183.8±37.5ms at a magnetic field strength of 1.5 T and 3T, respectively.^25^

### Extracellular Volume Fraction (ECV)

Native (pre-contrast) and post-contrast T1 mapping can also be used for the calculation of extracellular volume fraction (ECV). Unlike native T1 relaxation times, contrast-enhanced T1 values are more variable and dependent on contrast agent dosing, the time interval between contrast administration and measurement, and renal clearance. The estimation of the ECV (interstitium and extracellular matrix) requires measurement of myocardial and blood T1 before and after administration of contrast agents as well as the patient’s haematocrit value according to the formula:

ECV represents a physiological parameter and is more reproducible between different magnetic field strengths, vendors, and acquisition techniques than either native or post-contrast T1 mapping.^28^ ECV measures also exhibit better agreement with histological measures of the collagen volume fraction than isolated post-contrast T1 mapping.^29^ Normal ECV values of 25.3±3.5% have been reported in healthy individuals at a magnetic field strength of 1.5T.^26^ Apart from deposition of amyloid fibrils in the extracellular space, an increased ECV is most often due to excessive collagen deposition as in systemic sclerosis,^26^ and therefore represents a more robust measure of myocardial fibrosis. Low ECV values occur in thrombus and fat/lipomatous metaplasia. ECV can be calculated either from myocardial regions-of-interest or visualized on ECV maps similar to T1 and T2 mapping.^27^ Using this approach, ARD patients were found to have higher T1 and T2 values, as well as expanded ECV compared to controls, with most significant differences between native T1 and T2, which seem to be independent of the presence of LGE.^30^ Furthermore, native T1 mapping is sensitive to myocardial oedema, iron overload, and the presence of myocardial infarcts and scarring,^19^ and allows to follow longitudinal changes during treatment trials.^19^

### CMR in patients with ARDs

In general, the limitations of CMR examinations are centred around their high costs and time-consuming nature, both of which limit its everyday applicability in general cardiology. However, the impressive yield of CMR examinations with regard to the diagnosis of silent cardiac involvement that is often missed by other imaging modalities has been recognised in recent practice guidelines.^31,32^ Particularly for patients with ARDs, a CMR protocol including biventricular function assessment, LGE, T1, T2 mapping, and ECV, which can be performed in less than one hour, can be proposed as a sufficient clinical tool for every day clinical practice. If there are other queries, such as valvular disease quantification or vascular assessment, then other more sophisticated approaches should be added. It should be kept in mind that a CMR examination should be individualised according to the clinical scenario of the individual patient, and not performed uniformly as a one-size-fits-all approach in all ARD patients, since this might increase scanning time without necessarily answering providing additional information regarding the reason of referral.

To summarise, although no practice guidelines currently provide specific indications for a CMR examination in patients with ARDs, the authors recommend that a CMR examination should be considered in the following cases:

1) If there is a mismatch between patient symptoms and results of blood and/or imaging biomarkers;
2) In cases of new-onset HF and/or arrhythmia;
3) If the patient does not respond adequately to the immunomodulatory treatment;
4) If the underlying disease is quiescent, but the patient has cardiac symptoms; or
5) At the time of diagnosis for patients with systemic sclerosis and systemic lupus erythematosus, since cardiac involvement may be present and require immunomodulatory intervention, even if the systemic signs of SSc or SLE are minimal.

## CONCLUSION

Until recently, the evaluation of cardiac involvement in patients with ARDs was based on the presence of cardiac symptoms and the assessment of cardiac functional changes that only manifest as late findings. Currently, cardiac tissue characterisation using CMR allows for the early and robust identification of pathophysiologic phenomena that take place before clinically overt cardiac disease can manifest. As such, CMR provides considerable diagnostic utility and can inform early clinical decision-making with regard to appropriate immunomodulatory therapies. These in turn permit the individualization of patient treatment, ultimately leading to a truly personalized medicine.
